# Range bagging: a new method for ecological niche modelling from presence-only data

**DOI:** 10.1098/rsif.2015.0086

**Published:** 2015-06-06

**Authors:** John M. Drake

**Affiliations:** Odum School of Ecology, University of Georgia, 140 E Green Street, Athens, GA 30602-2202, USA

**Keywords:** ecological niche model, niche, Qhull, range, species distribution model, zero net growth isocline

## Abstract

The ecological niche is the set of environments in which a population of a species can persist without introduction of individuals from other locations. A good mathematical or computational representation of the niche is a prerequisite to addressing many questions in ecology, biogeography, evolutionary biology and conservation. A particularly challenging question for ecological niche modelling is the problem of *presence-only modelling*. That is, can an ecological niche be identified from records drawn only from the set of niche environments without records from non-niche environments for comparison? Here, I introduce a new method for ecological niche modelling from presence-only data called *range bagging*. Range bagging draws on the concept of a species' *environmental range*, but was inspired by the empirical performance of ensemble learning algorithms in other areas of ecological research. This paper extends the concept of environmental range to multiple dimensions and shows that range bagging is computationally feasible even when the number of environmental dimensions is large. The target of the range bagging base learner is an environmental tolerance of the species in a projection of its niche and is therefore an ecologically interpretable property of a species' biological requirements. The computational complexity of range bagging is linear in the number of examples, which compares favourably with the main alternative, Qhull. In conclusion, range bagging appears to be a reasonable choice for niche modelling in applications in which a presence-only method is desired and may provide a solution to problems in other disciplines where one-class classification is required, such as outlier detection and concept learning.

## 1. Introduction

The aim of ecological niche modelling is to construct a mathematical or computational representation of the environmental tolerances of a species and/or the species potential spatial distribution based on those tolerances. Niche modelling that uses only data about the environment at locations where the species is found (called *occurrence records*) is referred to as the problem of *presence-only modelling* [1–3]. The predictive performance of presence-only models is typically poorer than that of methods that aim to optimally discriminate locations where species are present from locations where they are absent [4,5]. For this reason, *presence–absence* modelling is sometimes preferred when additional data are available that reliably may be scored as absences [4]. Alternatively, *presence–background* modelling aims to discriminate the set of environments occupied by a species from the background distribution of environments from which these are selected [6,7]. Presence–absence and presence–background approaches both assume that the modelling objective is statistical classification of examples of two classes of environments (i.e. niche versus non-niche environments or niche versus background environments).

The use of classification methods for ecological niche modelling is controversial, however [4,8,9]. One reason is that sampling from the distribution of niche environments is difficult. The set of environments constituting the niche is well defined. The niche is the set of environments in which a population of the species could persist (subject to other conditions, such as that it is introduced in sufficient numbers to overcome Allee effects and it is not excluded by interactions with other species [10,11]). However, only if the species is at equilibrium within its range, all niche environments are realized in nature, detection probability is uniform across environments (i.e. detection probability is independent of local abundance, habitat type, distance to roads, etc*.*), and there are no occurrences of the species at places it cannot persist in the absence of immigration (i.e. the number of records of the species in population *sinks* is negligible [12]) may one assume that occurrence records are drawn from the distribution of niche environments in nature. These biases often prevent random sampling from the distribution of niche environments or even correcting a non-random sample [11,13,14].

Similar problems prevent sampling from the density of non-niche environments, where the problem may be exacerbated. Again, the definition is clear enough: non-niche environments of a species are those environments (real or imaginary, compare reference [15]) that are not within the set of environments in which a population of the species could persist in isolation from other sources. The spatial distribution of these in nature is also well defined. It is the locations of all such environments among the realized environments in nature. The problem is how this distribution is sampled and how that sample (together with a sample from the niche environments) relates to statistical classification. For instance, while the space of habitable environments is relatively small, the number of ways that an environment may be uninhabitable is huge (and, of course, most of these will not be realized in nature to be sampled from). The ambiguity of the classification problem introduces additional sampling biases. Are absence locations chosen to be those at which searches for the species were made but nothing collected or from all locations? But, even where searches have been made, these will be of different intensities at different locations and in any case will not be distributed in the same way as the non-niche environments themselves. Or, should absence locations be taken to be those that are ‘geographically close’ to occurrence records, i.e. locations that the species presumably had opportunity, but failed, to colonize (the set of ‘migratorily accessible’ environments *M* in the framework of Peterson *et al*. [11])? Or, should absence locations be taken to be those that are ‘environmentally close’ to occurrence records, i.e. environments that might best delineate the boundary of the niche in ecological space? These problems are well known in ecology [11,16], and, for reasons like these, together with the impossibility of documenting non-occurrence in a non-exhaustive sample, and the expense and difficulty of obtaining sufficiently large samples to determine that even if a species is present at a site it must be present in low numbers, many studies have ceased aiming to discriminate presences from absences, but only to discriminate presences from the joint distribution of environments overall (presence–background models) [7].

Unfortunately, there are even more problems. There are numerous reasons why species are not found in environments in which they could persist (even granting the assumptions of range equilibrium and migratory accessibility), including species interactions, metapopulation dynamics and disturbance cycles [10]. For some species, it is plausible that the *majority* of the locations within a species' niche will not be occupied [17]. Indeed, as the spatial resolution at which sites are delineated increases, the fraction of sites occupied will typically decline, so that prevalence is a scale-dependent property [18]. Thus, regardless of whether a presence–absence or presence–background approach is taken, from a practical point of view, the occurrence records will always be a subset, not a contrasting class. For these reasons, presence-only methods might be preferred for the development of species distribution models as well as other applications of niche modelling [4,19].

### 1.1. The goal of niche modelling

**Figure 1.**
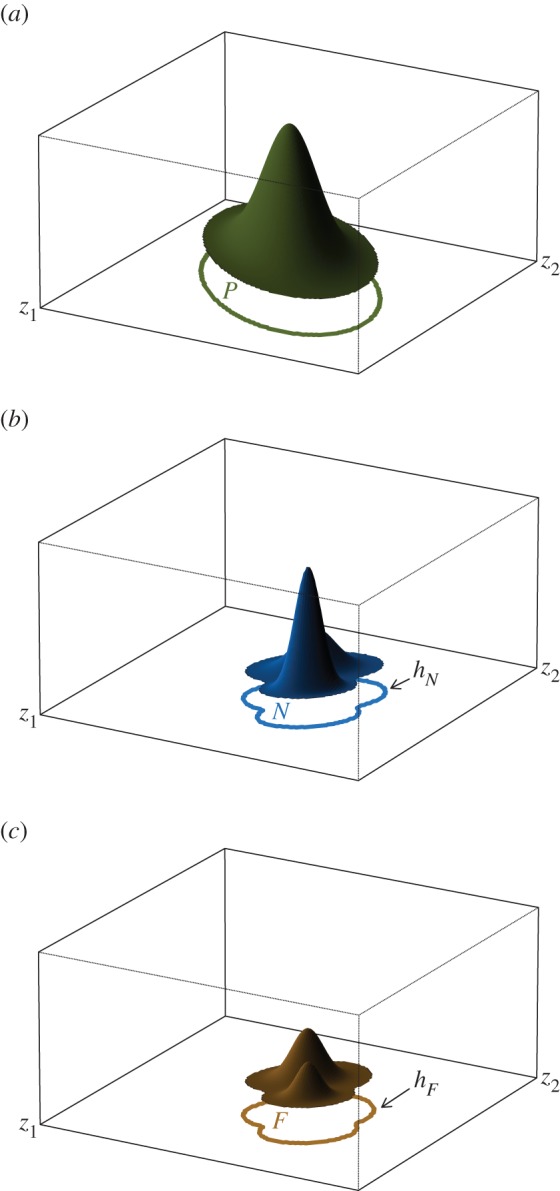
The probability density of environments occupied by a species, *f*(*z*), is the product of their distribution in nature, *p*(*z*), and the habitat selection function, *q*(*z*). *P* is the set of environments realized in nature. The boundary *h_N_*(*z*) separates niche environments, *N*, from non-niche environments. A similar boundary, *h_F_*(*z*), encloses the support *F* of the distribution of environment in nature. If *P* is broad with respect to *N*, then *h*_*N*_(*z*) ≈ *h*_*F*_(*z*) even if *p*(*z*) and *q*(*z*) are far from uniform. (*a*) Probability density of environment *p*(*z*). (*b*) Habitat selection function *q*(*z*) > 0. (*c*) Density of occupied environments *f*(*z*) = *p*(*z*)*q*(*z*).

There is also a positive case for presence-only modelling. Central to the idea of the niche is the ecological concept of *tolerance* [10,20]. We start with the joint distribution of environments in nature *p*(*z*), *z* ∈ ℝ^*x*^ (see table 1 for a summary of notation; figure 1*a*). The set of all realized environments is designated *P* (although the picture in figure 1*a* is slightly misleading—*P* may not be simply connected). We will say that a species tolerates an environment *z* if and only if it can locally persist (i.e. persist in the absence of supplemental migration) in *z*. The effect of environment *z* on the persistence of a population is determined by the fitnesses of individuals in that environment. We will assume that fitness in *z* may be mapped to a *habitat selection function*, *q*(*z*), which gives the probability that environment *z* is occupied by the species (figure 1*b*) [21,22]. We assume *q*(*z*) = 0 if and only if fitness in *P* is less than one. The niche is defined by the indicator function1.1
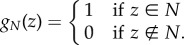
The limits to tolerance are the boundaries in the environmental space between the subsets of the environments in which the species can persist (the niche, *N*) and those where it cannot, or the *zero net growth isocline*, denoted *h_N_*(*z*), which is the boundary of *N* [23,24].

**Table 1.** Notation used in this paper.

symbol	definition
variables	
*z* ∈ ℝ^*x*^	the environment, a vector of environmental variables
*x*	dimension of environment
*δ*	order of the range-bagging model
*v*	number of votes used for the range-bagging model
*n*	number of occurrence records
*X_i_*	bootstrap sample
*w*	tuning parameter
*ε*	tuning parameter
*p* = *w*/*n*	fraction of occurrence records used in the bootstrap sample
*k*	number of points in the convex hull
functions	
*p*(*z*)	probability density of environments
*f*(*z*)	probability density of environments occupied by species
*q*(*z*)	habitat selection function
*g_N_*(*z*)	mapping from environmental space to niche
*h_N_*(*z*)	zero net growth isocline, the boundary of the niche
*h_F_*(*z*)	boundary of the set of occupied environments
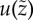	niche centrality
sets	
*P*	set of realized environments
*F*	set of occupied environments
*N*	niche, the set of environments in which a species can persist
Δ*_*δ*_*	marginal niche, a projection of *n* onto a *δ*-dimensional subspace Δ ⊂ *N*
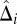	marginal niche model for the *i*th bootstrap sample

**Figure 2.**
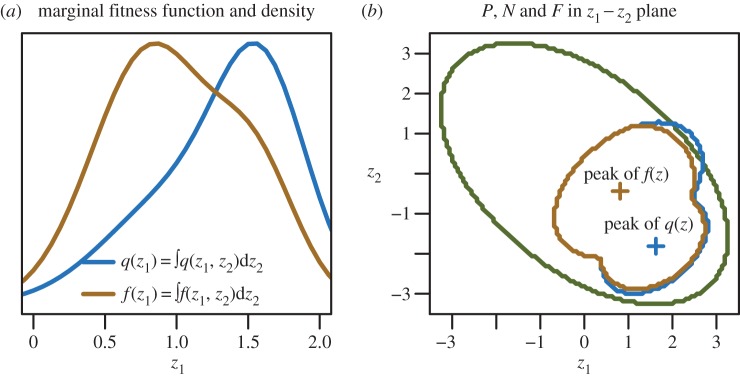
If the density of environments *p*(*z*) is far from uniform, the distribution of occupied environments in nature, *f*(*z*) may bear little resemblance to the habitat selection function *q*(*z*). This plot shows the two-dimensional habitat selection function, *q*(*z*), and joint density of occupied environments, *f*(*z*), ‘marginalized’ over variable *z*_2_ (*a*). Importantly, the maxima of these functions are displaced from each other by approximately half the habitable range. Nonetheless, *h_F_*(*z*), the boundary of the support of *f*(*z*) may be a very good approximation to *h_N_*(*z*), the zero net growth isocline (*b*). Note, particularly, that even though the maxima of *p*(*z*) and *q*(*z*) belong to different modes the supports of these functions are nearly identical.

I suggest that we think of *niche identification* as the estimation of *h_N_*(*z*). Obviously, the distribution of occupied environments in nature, *f*(*z*), depends on both the density of environments from which species can select and the habitat selection function (figure 1*c*). We designate this set *F* and denote its boundary by *h_F_*(*z*). The key insight is that if the set *P* is ‘large’ compared with *N*, then *h*_*N*_(*z*) ≈ *h*_*F*_(*z*) and a model of *h_F_*(*z*) may be substituted for *h_N_*(*z*) in practice. Figure 2 presents this idea graphically. What it means for *P* to be large is somewhat ambiguous. The intuition is that information is required mainly near the boundary of *N*, the zero net growth isocline and is relatively unimportant elsewhere. Possibly, this criterion could be made more precise by stating additional conditions ensuring that the species had the opportunity to explore its environmental space, for instance that for all points in *h_N_*(*z*) there must exist within a local neighbourhood points in *P*. Importantly, the approximation of *h_N_*(*z*) by *h_F_*(*z*) may be good even where *f*(*z*) and *q*(*z*) have very different shapes (figures 1 and 2). This is useful because one typically has data drawn from *f*(*z*) but not *q*(*z*). For this reason, we may wish to speak of ‘estimating the support of *f*’, by which we mean estimating the parameters of a model 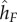, or a trained algorithm. The estimation of *h_F_* may be construed as a classification problem, but does not have to be. Further, this picture makes no explicit assumptions concerning the *prevalence* of a species in nature (i.e. whether *q*(*z*) is large or small in places where it is positive).

The framework introduced here assumes that (i) the realized environments (*P*) are large with respect to the species niche, (ii) the environmental space has been widely sampled by the species (compare [16]) and (iii) few occurrences are found in sink environments (i.e. there are few occurrence records where, in fact, *q*(*z*) = 0). While this picture differs from that of many authors with respect to the proper goals of niche modelling, assumptions (i)–(iii) are common to virtually all approaches. Finding methods that achieve the objectives of niche modelling when these assumptions are violated is an important area for further work. Concerning (ii), particularly, although we require *N* ∩ *P* ≠ Ø we do not require *N* ⊂ *P* and we do not require the species to have ‘sampled’ *P* in any particular way (i.e. randomly, or at spatial equilibrium, or evenly—although we do want it to have sampled a wide range of *P*, particularly in the vicinity of the unknown boundary *h_N_*). For instance, in a continuous niche dimension (e.g. average annual temperature), the fundamental niche may consist of a closed interval [−1.75, 2.75] as for variable *z*_1_ in figure 2. By hypothesis (and contrary to the illustration in figure 2), we assume that no locations presently exist with (*z*_1_ = 2.2, *z*_2_ = 2), so that this environment is not in the support of the sampling density *f*. Nevertheless, because this point is contained in *N* (and enclosed by the boundary *h_N_*), it may nevertheless be included in the estimated niche. A trickier problem is presented by the subset of *N* not contained in the boundary of *P*. In figure 2, these are environments in *N*\*P*. Inevitably, this kind of failure leads to biased estimates of the boundary *h_F_*. The point of figure 2 is that this bias may not be severe (the maximum displacement between *h_F_* and *h_N_* is much smaller than the displacement between the peaks of *f*(*z*) and *q*(*z*) in figure 2). However, there are no guarantees. The conclusion of this argument is that numerical methods that aim to model the support of a distribution [25–28] may work much better for niche modelling than methods that focus on matching the higher moments (mean, variance, etc*.* [8,6,29]). At the very least, such methods could enrich the niche modelling toolbox.

### 1.2. What is a range?

The main objective of this paper is to propose a new method for ecological niche modelling called *range bagging*. Range bagging is motivated by the success of popular ensemble methods for ecological niche modelling (e.g. boosted regression trees [30]) together with a closer look at what ecologists mean by ‘niche’. The conception that I propose is that *the niche is the range of environments in which the species can persist*. But, what is a range? In general, range is the interval between two extremes of an ordered set. In biogeography, range is the interval between two extreme occurrences (e.g. *latitudinal range*, compare [31,32]). In statistics, range is the interval between the minimum and the maximum of a sample [33]. In some cases, we do not have an ordered set, but rather consider range to be the set of possibilities or the cardinality of the set. In parasitology, *host range* is the number or composition of host species infected by a parasite [34]. In mathematics, range is the set of all values of a function, i.e. its image [35].

Broadly in keeping with these related concepts of range, we will say the environmental range of a species consists of the closed interval defined by its tolerance limits (minimum and maximum) for an environmental variable (e.g. temperature [36]). To extend this concept of range to multi-dimensional environmental spaces, we will say that the environmental range of a species in ℝ^*x*^ is the smallest convex set that contains *N*, i.e. the convex hull of *N*. A set is convex if and only if for every pair of points within the set, every point on the straight line segment joining the pair is also within the set. Defined this way, the environmental range satisfies our intuitions about what a range is and, moreover, is invariant to translations and rotations of the environmental coordinate system. Equivalently, the convex set contains the univariate ranges of all possible rotations of the environmental coordinate system.

## 2. Methods

### 2.1. Range bagging

While it is unknown if species' niches typically are convex and simply connected, it is plausible that they might be. (Under what conditions would species evolve non-convex, non-connected niches?) Although non-convex niches have sometimes been measured [37, p. 114], both classical [38, p. 235] and contemporary [8,39,40] contributions often assume niches to be convex. In any case, niches with irregular, complicated boundaries are likely to be rare and a convex, simply connected space appeals as an approximation to something more complicated. It follows that if an approach can be developed to estimate species environmental ranges from data, such a model might also be interpreted to be a model of the niche. Range bagging is such an approach.

The basic idea of range bagging is to vote ranges of environmental variables obtained from bootstrap samples of a sample from the distribution *f*(*z*). To explain in more detail, we first introduce the concept of the *marginal niche*, which is the environmental range of a species viewed from a lower-dimensional perspective. Specifically, a set Δ*_*δ*_* is a marginal niche if and only if it is a *δ*-dimensional projection of *N* onto a space Δ ⊂ *N* of dimension *δ* < *x*. If *δ* = 1, the marginal niche is the numerical range of an environmental variable over which the species may be found, say salinity or temperature. Following the discussion above, the concept of marginal niche is readily extended to dimension *δ* > 1 as the convex hull in ℝ^*δ*^ of the environments in *N*. The value of *δ* is central to the range-bagging algorithm. We say that *δ* is the *order* of the model.

One (possibly unrealistic) proposal is to use the *x*-dimensional convex hull of the occurrence records as a model of the niche [41,42]. This idea is unworkable, because convex hulls in high dimensions are typically too complex to compute (the upper bound theorem gives the worst-case complexity as *O*(*n*^⌊*δ*/2⌋^), where ⌊ · ⌋ is the floor function [43]) and because typical samples (at best drawn evenly with respect to the individual environmental variables and more likely with some substantial tendency to be concentrated) will be highly clustered in the centre of a high-dimensional space. A second proposal, then, is to use an estimate of the marginal niche, comprised of the convex hull of a subset *δ* < *x* of the original environmental variables, as a reduced or partial model of the niche. Given that we are thinking of the marginal niche as a projection of *N* onto Δ, we might think of this as the niche from a particular *perspective*, say from the *perspective of thermal tolerance* or from the *perspective of available food resources*. Such a model is expected to have better statistical properties than a model constructed from the convex hull of the entire set of occurrence records, because the range is more evenly sampled in *δ* dimensions than *x* dimensions.

However, we can possibly do even better. It has recently become apparent in many disciplines, including ecology, that ensembles of models are often more reliable (stable, accurate, unbiased) than individual models. In machine learning, one approach to ensemble modelling is called bootstrap aggregation or *bagging* [44]. Bagging consists of multiply selecting a bootstrap sample of the original data, fitting models to individual samples and averaging the outcome [45]. Range bagging, then, consists of two core steps:

let *n* be the number of records in the dataset, represented by *x* environmental variables. For each iteration, *i* ∈ [1,2,3, … *v*];

*(1) Sample step.* For a model of order *δ*, randomly select (without replacement) *δ* < *x* environmental variables. From the resulting *n* × *δ* table of records, randomly select (without replacement) *w* ≤ *n* records to be included in the bootstrap sample, *X_i_*.
*(2) Marginal niche estimation step.* As a *base learner*, estimate the marginal niche 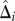 of the points in *X_i_*. If *δ* = 1, the marginal niche is simply the interval between the minimum and maximum values in the bootstrap sample. For *δ* > 1, the marginal niche is the convex hull of the bootstrap sample.

A new point 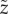 is assigned *niche centrality* 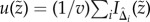, where *I*(·) is the indicator function. That is, the point 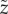 is tested for whether it belongs to each of the estimated marginal niche models 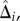 and the resulting ensemble of predictions is averaged to provide an index. In the event that we seek a categorical response (niche/non-niche), class assignment will be made using the indicator function 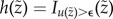 for some 0 ≤ *ε* ≪ 1. Note that a choice of *ε* > 0 implies the species to have been found in a sink environment.

A couple of notes on this procedure are in order. First, for the bootstrap samples, it does not make sense to sample with replacement, because duplicated points have no effect on the estimate of the range (minimum and maximum) or the convex hull of points. Second, the value *w* is a tuning parameter (perhaps best thought of in terms of the fraction of points sampled, *p* = *w*/*n*). This model will be increasingly ‘conservative’ (in the sense of excluding peripheral but tolerable environments from the estimated marginal niche 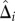) as *p* is reduced from its maximum at one towards zero. This could be very useful with species or datasets in which a lot of examples are from sink habitats. In this respect, *p* may be thought of as a robustifying parameter. In the typical case where it is assumed that observations from sink habitats are rare or non-existent, we will set *p* = 1 to maximize the chance of sampling the extreme cases that are most informative about the niche boundary. Finally, range bagging has time complexity of the order of the base learners (*O*(*n*) for *δ* = 1 and *O*(*n* log *k*) for *δ* = 2 and *δ* = 3, where *k* is the number of points in the resulting hull).

### 2.2. R implementation

The range bagging algorithm is easily implemented in R [46] using the interface provided by the geometry package to the Qhull library [47]. This study was performed using only two new functions (rb and rb.test) that provide wrappers to the functions convhulln for computing the convex hull of a multi-dimensional set of points and tsearchn for determining which element of a Delaunay triangulation a particular point belongs to, which may be used as a test for whether the point is contained within the convex hull. These functions are included in the electronic supplementary material.

**Figure 3.**
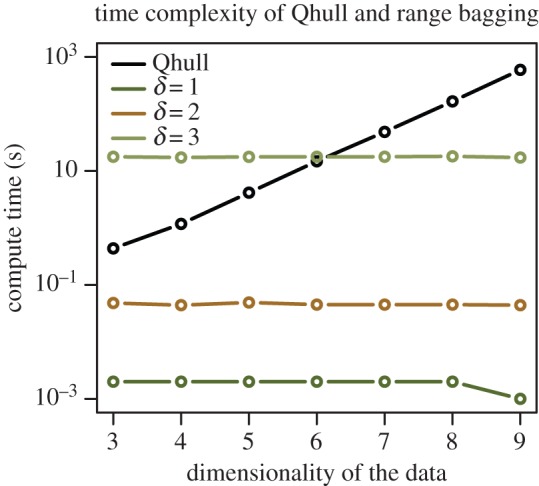
Time to fit and test *n* = 100 points using the Qhull algorithm increases exponentially with *d* ∈ [2, 3, 4, … 9] dimensions. Time to fit range bag models of orders *δ* ∈ {1, 2, 3} is *O*(*n*).

To compare computational complexity with Qhull (the full convex hull), I simulated *n* = 100 training records from a *d*-dimensional (*d* ∈ [1, 2, 3, … 9]) multivariate normal distribution with mean 0 and unit variance. These were considered to be a sample of occurrence records from the joint distribution *f*(*z*). Next, I generated a sample of 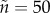 test records from a *d*-dimensional multivariate normal distribution with mean 5 and unit variance. Computing time (time required to test 50 records selected from the original 100 used for training together with the 50 outliers) for range bag models was almost constant with dimension, whereas time required to compute the full convex hull with Qhull increased approximately exponentially (figure 3).

### 2.3. Case study: two-spined blackfish

**Figure 4.**
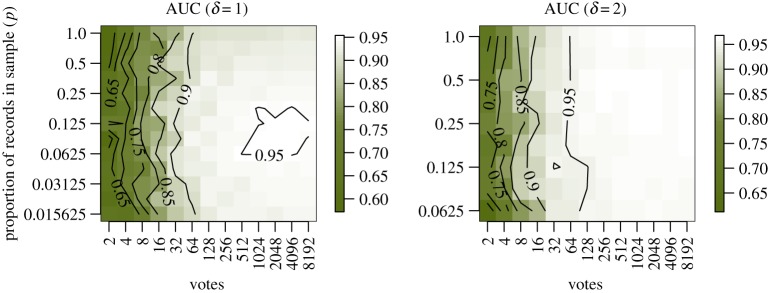
Performance of *δ* = 1 and *δ* = 2 range bagging over a range of *p* (the proportion of records included in bootstrap samples) and *v* (the number of base learners or votes). In the *δ* = 1 case, AUC is maximized at an intermediate value of *p*. In the *δ* = 2 case, there is no evidence for this dependency, where AUC increases monotonically with *v* reaching its maximum around *v* = 128.

To illustrate, I compared range bagging with MaxEnt using data from a case study on two-spined blackfish *Gadopsis bispinosus*, a medium-sized (15–17 cm length) freshwater fish that inhabits flowing waters of Australia's Murray–Darling river system. Data are from Elith *et al.* [7] and include occurrence records for 255 sites and background data from a random sample of 10 000 of the approximately 240 000 mapped river segments within the biogeographic range of *G. bispinosus*. Covariate data comprised 19 variables pertaining to climate, geography and ecology at three spatial scales as well as an indicator variable for the subriver system to which each segment belongs. Elith *et al.* [7] fit a MaxEnt model to these data and obtained an average AUC of 0.97 on withheld data in cross-validation. To look at the performance of range bagging with respect to the tuning variables, I performed one- and two-dimensional range bagging (*δ* = 1 and *δ* = 2) on the occurrence data over a range of the tuning variables *v* (number of base learners voted to obtain the final model) and *p* (the fraction of records in each bootstrap sample). To measure performance, I computed the average AUC in 10-fold cross-validation. The results may be visualized as a heat map (figure 4). For *δ* = 1, range bagging achieved a maximum AUC of 0.954 at *p* = 2^−7/2^ ≈ 0.088 and *v* = 1024. For *δ* = 2, the maximum AUC was 0.968 at *p* = 2^−3/2^ ≈ 0.354 and *v* = 256, indistinguishable from the MaxEnt result reported by Elith *et al.* More importantly, the increase in performance increased rapidly with the number of votes reaching its maximum quickly and showing no evidence for a reduction with further increases in *v*. Further, as clearly shown in figure 4, there was very little effect of *p* on the performance at all. Together, these observations suggest that range bagging, like other ensemble methods, may be deployed in a way that is very robust to model choices.

## 3. Discussion

### 3.1. Niche theory

What is the aim of niche theory? In my view, a theory of the ecological niche is successful if it provides the concepts needed to understand the relationship between species and their environments and accurately predicts the spatial distribution of species by way of these concepts. Although seemingly similar in intent, the framework adopted in this paper is different to that of Peterson *et al.* [11] in several respects. Consistent with standard practice in ecology, both assume that environments are represented by a vector of measurements (denoted *z* here and *e* by Peterson *et al.* [11]). However, *z* is restricted to the real numbers, whereas *e* in Peterson *et al.* is also intended to encompass categorical and ordinal covariates. The concept of environmental range developed here is not consistent with categorical and ordinal covariates. Moreover, most of the key concepts in the theory of Peterson *et al.* [11] are defined only with respect to existing environments, whereas the niche theory underlying range bagging applies to both realized and possible environments. Thus, for example, the set of realized environments (*P*), is central to range bagging, but appears only briefly in an appendix of Peterson *et al.* [11] (where it is designated by *N*). Similarly, the range bagging approach considers the niche (*N*) to be fundamental (and defined via *q*) and estimable, whereas Peterson *et al.* [11] are largely concerned with only the intersection of *P* and *N*. Further, range bagging considers the boundaries *h_N_* and *h_F_* to be the key targets of estimation. These quantities have no counterpart in the theory of Peterson *et al.* [11]. These differences are important for both practical and theoretical reasons. Practically, the two approaches lend themselves to different approaches to evaluation. A model of the environmental range is good when the fit model 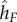 lies close to the unknown boundary *h_N_*. In their approach to evaluation, Peterson *et al.* [11] are primarily concerned with the correct prediction of species' occurrence (or conditional probability of occurrence) at observed locations, rather than at discrepancies between the fit model and the outcome of a hypothetical test (if the species were to be introduced to environment *z*, would it persist or not?). It is my view that the empirical assertions made by this latter subjunctive conditional statement are the essential characteristics of a niche theory [9].

### 3.2. Range bagging

This paper introduces range bagging, a new method for identifying the niche of a species from records of its occurrence in nature. Through simulation, I established that this method is more efficient (less computuationally complex) than fitting the full multi-dimensional convex hull. Although motivated by, and designed for, the ecological problem of niche identification, it is possible that range bagging could be useful for many kinds of one-class classification [25], including estimating the support of a statistical distribution [26], concept learning [48] and outlier detection [49].

Why does range bagging work? There is a theoretical worry. Although range bagging is not itself a bootstrap estimator, when *p* < 1 it does depend on bootstrap estimates of distributional minima and maxima. These are not theoretically consistent. (Estimates obtained in this way do not converge in probability to the true values.) Is this consistency a problem? Possibly not. Bagging algorithms are not generally consistent, although bagging preserves the consistency of its base learners, and may even convert inconsistent base learners to consistent ensemble learners [50]. Moreover, bagging may perform very well in applications even when inconsistent [50]. Motivated by this observation, it is easy to envision a number of extensions to the basic range bagging algorithm presented here and plausible that further gains in performance could be achieved. First, because the minimum and maximum of a sample (or of a bootstrap sample of a sample) are biased estimators of the true minimum and maximum of a distribution, the marginal niche models that are the base learners of range bagging will always be proper subsets of the models that would be obtained from an infinite sample, even if the ‘bootstrap’ sample contains the entire set of observed records. A variant of the original algorithm might seek to counter this bias by substituting an alternative estimator, thereby ‘stretching’ the boundary of the estimated base model. In the one-dimensional case, this might be achieved using an ‘average gap’-type correction [51] or by defining a small quantity, possibly a fixed fraction of the observed range, and extending the range by subtracting this quantity from the minimum and adding it to the maximum. In the higher-order (*δ* > 1) case, some principled means of extending the observed range would be required. Alternatively, extreme value theory might be used to parametrically put limits on the extrema [52]. Finally, because range bagging is ultimately based on the physiological tolerances of species, experimental data could be incorporated, perhaps by supplying a prior probability on the minimum or maximum or even stipulating a lethal conditions at which *q*(*z*) = 0 [53]. This would be particularly useful in cases where a species is suspected of not reaching the extents of its environmental tolerances for contingent biogeographic or ecological reasons (e.g. an invasive species not at range equilibrium [54]).

Another way in which range bagging needs to be extended is to allow for categorical environmental variables. Importantly, categorical variables, particularly unordered variables, would require that we change our concept of range. Specifically, a more general concept of connectedness is required to replace the simple connectedness assumption that allows us to define range in a multi-dimensional sense. This is a key issue for further theoretical development.

Besides asking how range bagging may be extended, it is interesting to consider how it is related to existing methods for machine learning. Particularly, range bagging is very similar to the method of random forests [44], particularly random decision ‘stumps’ (bagged decision trees consisting of a single binary classification). Indeed, range bagging was itself inspired by asking how a random forest could be used for one-class classification. Essentially, a decision stump for a density estimation problem is asking whether or not a test point is found in the range of a data sample. This idea led to the one-dimensional range bagging algorithm. The general approach (convex hulls) evolved naturally from inquiring what a multi-dimensional ‘range’ might be.

Finally, there are questions about how range bagging will perform under real-world data sampling scenarios. For illustration, I used range bagging in a case study of *G. spinosus*, where data are openly available and include a large number of covariates. Possibly, the extremely good performance of both MaxEnt and range bagging attests not only to the flexibility of these models, but also to the high predictability of this particular dataset. Nonetheless, the example shows that range bagging may perform comparably to other widely used methods. An interesting question (both for MaxEnt and for range bagging) is how well these methods perform in the presence of irrelevant variables. I expect that range bagging may result in poorly calibrated models (because lots of bootstrap samples would contain *only* irrelevant variables), but that these would not affect model discrimination, i.e. the rank ordering of environments by the model ensemble [3]. A related question is how best to select variables, particularly for range bagging (MaxEnt has *L*_1_ regularization built in, which can be used for variable selection [29]), and whether or not dimensional reduction through variable selection can yield stabler or more accurate models, or models that are better suited to guiding future studies by improving interpretability.

### 3.3. The goals of niche modelling

The introduction to this paper presented an argument about the goal of ecological niche modelling. Specifically, I argued that models of the ecological niche that aim to estimate the zero net growth isocline should more accurately represent the potential and actual distributions of a species than models that are aimed at fitting the central moments of the distribution of occupied environments. This argument assumes that the probability of habitat selection is greater than zero if and only if average individual fitness exceeds one. The relationship between fitness and habitat selection is an important area for further conceptual clarification, theoretical development and empirical testing.

I further argued that if the range of realized environments is broad with respect to the ecological niche, then boundary estimation methods for ecological niche modelling may be robust to sampling biases and awkward data distributions that are common in occurrence data. This breadth requirement does not entail that the niche environments must be a strict subset of the realized environments, although such a condition of strict nestedness would be sufficient. Particularly, there are two ways in which niche environments may not be realized (i) there may be ‘interior’ environments within the range (convex hull) of realized environments that simply do not exist and (ii) the niche may include environments that are outside the range (convex hull) of any conditions realized in nature. How these two violations of nestedness differently affect model fitting is an important problem for further consideration. Particularly, I would guess range bagging to be more robust to missing data of the first kind than other common methods, particularly those that are most flexible like boosted regression trees. It is expected that missingness of the second kind will affect the extrapolability of range bagging much more than violations of the first. In such cases, it is possible that parametric methods, which tend more to be ‘global fits’ to the data would be superior. However, in my view, whether or not *any* niche modelling methods are robust to missingness of the second kind is a very interesting question. These principles for ecological niche modelling are general and apply regardless of whether range bagging, the approach introduced here, is generally successful or not.

### 3.4. Are the niche and the environmental range equivalent?

I conclude with a new question: are the niche and the environmental range of a species equivalent? Certainly, all environments in which a species can persist (all the environments in its niche) are elements of its environmental range, by definition. But, are all the environments of a species' range also environments in which it can persist? This is true only if the niche is convex and simply connected. The definition of the environmental range presented here, the convex hull of the environments tolerable to a species (the environments in which it can persist), allows this to be an empirical, rather than merely conceptual, question.

## Supplementary Material

R functions for range bagging
